# Boosting the Emission of Momentum Indirect Interlayer Excitons by an Optical Near Field in Misaligned 2D Heterostructures

**DOI:** 10.1021/acs.nanolett.5c02703

**Published:** 2025-10-07

**Authors:** Qixing Wang, Takashi Taniguchi, Kenji Watanabe, Jurgen H. Smet

**Affiliations:** † Department of Physics, College of Physical Science and Technology, 12466 Xiamen University, Xiamen 361005, China; ‡ Jiujiang Research Institute, Xiamen University, Jiujiang 332000, China; § Max Planck Institute for Solid State Research, Stuttgart D-70569, Germany; ⊥ Research Center for Materials Nanoarchitectonics, National Institute for Materials Science, Tsukuba 305-0047, Japan; ∥ Research Center for Electronic and Optical Materials, 52747 National Institute for Materials Science, Tsukuba 305-0047, Japan

**Keywords:** van der Waals heterostructures, momentum mismatch, interlayer excitons, emission enhancement, optical near field

## Abstract

The moiré superlattice potential in van der Waals heterostructures localizes the interlayer excitons and modifies the band structure so that their emission wavelength can be adjusted substantially. However, twisted structures suffer from poor emission quantum yield and long radiative lifetimes due to the angle-induced momentum mismatch. Moreover, the vertical orientation of the interlayer exciton hampers the collection efficiency. Here, we demonstrate that these unfavorable conditions can be fully overcome by embedding the heterostructure in a plasmonic circular nanocavity. By adjusting the cavity radius, the emission enhancement factor for the interlayer excitons can exceed 4 orders of magnitude due to the synergistic effect of photon momentum enlargement and promotion of the excitation rate, quantum yield, and collection efficiency by the optical near field. This strategy for engineering light–matter interactions can make these atomically thin heterostructures as alluring as their direct-band-gap opponents in the field of optoelectronics.

## Graphical abstract

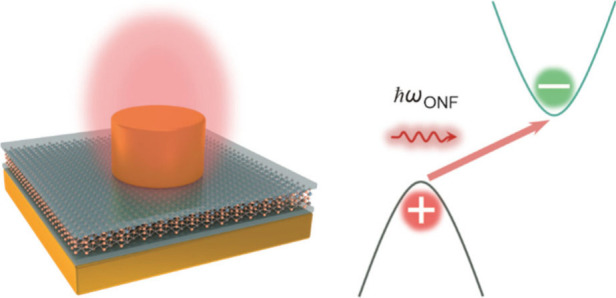

Optical absorption and emission in semiconductors need to satisfy the requirements of both energy and momentum conservation.^1^ In direct-band-gap semiconductors, absorption and emission can occur efficiently by direct interband transitions because the conduction band minimum (CBM) and the valence band maximum (VBM) are located at the same momentum in the Brillouin zone. In indirect-band-gap semiconductors, however, the momenta of the CBM and the VBM are distinct and the momenta of photons are typically 2–3 orders of magnitude smaller than the momentum mismatch.^1–3^ To compensate for the band momentum mismatch, phonons must get involved in the interband transition process (Figure 1a).^4^ Because optical absorption and emission now demand a three-particle interaction between photons, electrons, and phonons, their efficiency drops significantly.^4,5^ Hence, indirect-band-gap semiconductors, such as for example silicon, are not ideal for light-emitting device applications^5^ without additional measures, and a considerable effort has been devoted in the community to improve their optical absorption and emission efficiency.^6^ For example, by utilization of the Purcell effect of a compact plasmonic nanocavity, the PL intensity of momentum indirect interlayer excitons of a 30°-twisted MoS_2_/WS_2_ heterobilayer was enhanced over 2 orders of magnitude due to the Purcell effect.^7^ Here, we demonstrate that for van der Waals heterostructures with smaller twist angles a boost of 4 orders of magnitude is possible by exploiting the momentum offered by the optical near field (ONF) in the cavity.

**Figure 1:**
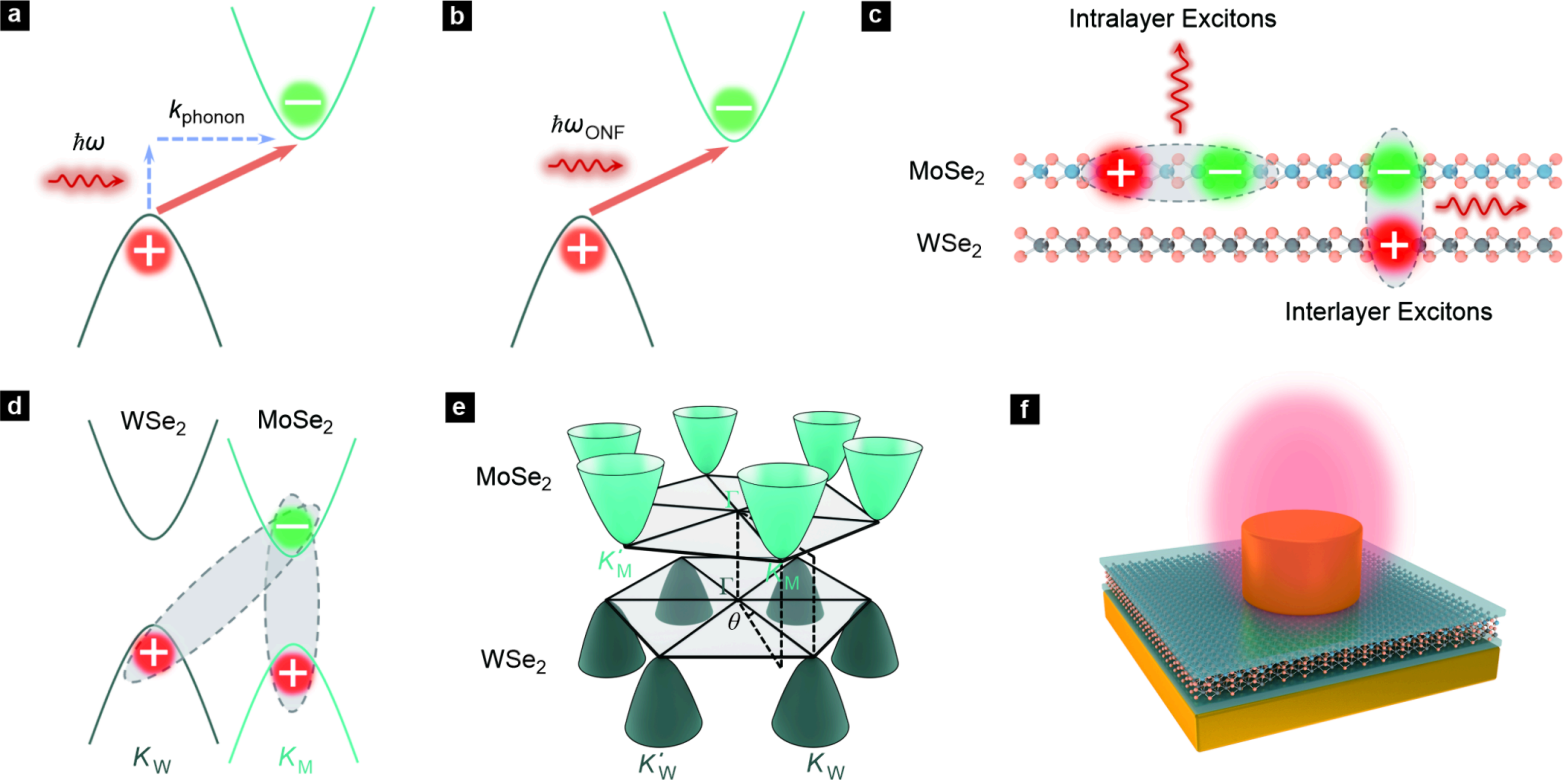
Momentum indirect interlayer excitons in a twisted WSe_2_/MoSe_2_ heterostructure. (a) Schematic illustration of the optical excitation process in an indirect-band-gap semiconductor. Such an excitation requires the interaction of photons, electrons, and phonons. (b) Illustration of direct excitation of an electron in an indirect-band-gap semiconductor without the assistance of phonons but with the help of photons with larger momenta from the ONF in a confined volume. (c) Schematic illustration of the dipole orientation of interlayer excitons in a WSe_2_/MoSe_2_ heterostructure and intralayer excitons in a MoSe_2_ monolayer. (d) Illustration of the direct nature of intralayer excitons in both real and reciprocal space as well as the indirect nature of interlayer excitons in both real space and reciprocal space for a twisted WSe_2_/MoSe_2_ heterostructure. The interlayer exciton is composed of a hole located near the *K*_W_ symmetry point in *k* space of WSe_2_ and an electron at the *K*_M_ point of MoSe_2_. (e) Band structure of a twisted WSe_2_/MoSe_2_ heterostructure. Only the conduction band edges of the MoSe_2_ monolayer and the valence band edges of the WSe_2_ monolayer are shown. As a result of the real space twist θ, also the Brillouin zone with the *K*_W_ and *K*_W_′ symmetry points of the WSe_2_ monolayer is rotated away by the same angle θ from the Brillouin zone of the MoSe_2_ monolayer. (f) Schematic of a plasmonic nanocavity consisting of a gold nanopillar placed on top of a gold film on the substrate and separated from this gold film by the van der Waals heterostructure composed of a multilayer of hBN, a WSe_2_ monolayer, a MoSe_2_ monolayer, and a multilayer of hBN. For the samples fabricated here, the heterostructure has a total thickness of about 7.5 nm.

Indeed, a viable strategy to promote the optical absorption and emission in indirect-band-gap semiconductors is to aggrandize the photon momentum, so that there is no longer a need for phonon participation. This can be accomplished by exploiting the consequences of the Heisenberg uncertainty principle for the position (*x*) and momentum (*p*) variables^8,9^ of the photon. When the photon is confined to a small volume, its position uncertainty Δ*x* is reduced and the momentum uncertainty Δ*p* gets enhanced accordingly: Δ*x*Δ*p* ≥ ℏ/2. The broadening of the momentum distribution of the photon outwits the momentum conservation rule and enables interband transitions in indirect-band-gap semiconductors without the assistance of phonons (Figure 1b).^2–5,10^ The two-particle interaction between the light in the ONF and the electron then guarantees a reasonable efficiency for optical absorption and emission also in indirect-band-gap semiconductors.^3–5,10–12^

Monolayer transition-metal dichalcogenides (TMDCs) are a burgeoning class of materials with strong light–matter interaction. This makes them appealing candidates for the application in optoelectronic and light-emitting devices.^13–18^ With the help of van der Waals stacking techniques, it is possible to generate vertical homo- or heterostructures with enticing optical properties^19–28^ that strongly depend on the twist angle between the layers. The twist angle can be estimated from polarization-dependent second-harmonic-generation measurements, as shown in Section S2.^29,30^ For aligned (0°) and antialigned (60°) van der Waals structures, strong interlayer coupling causes a hybridization of the electronic bands, and they behave as direct-band-gap semiconductors with large optical oscillator strength^31–36^ and in-plane interlayer exciton transition dipoles.^37^ In contrast, misaligned structures with twist angles away from 0° and 60° behave without any additional measures as indirect-band-gap semiconductors that exhibit low optical oscillator strength^31–33,38^ and show an interlayer exciton emission pattern that corresponds to an out-of-plane transition dipole.^39^ A specific example is shown in Figure 1d,e. They illustrate the momentum mismatch between the CBM of MoSe_2_ and the VBM of WSe_2_. Because the dipole formed by interlayer excitons is oriented perpendicular to the plane, its optical radiation propagates in the plane and is difficult to collect in reflected light microscopy. Despite these unfavorable conditions, it would be rewarding to overcome both the lack of oscillator strength and collection efficiency in these twisted structures. In this study, we demonstrate that indeed in such intentionally engineered indirect-band-gap systems intense optical emission can still be achieved from these interlayer excitons in a plasmonic nanocavity through photon confinement that is accompanied by an increase in the uncertainty of the photon momentum. A giant emission enhancement as large as 4 orders of magnitude can be achieved.

These studies were performed on a twisted WSe_2_/MoSe_2_ heterostructure sandwiched between a gold film and a hexagonal boron nitride (hBN) multilayer on one side and an hBN multilayer and gold nanopillar on the other side. A cross-sectional schematic of the device is depicted in Figure 2a. The hBN layers act as spacers between the gold film and the gold nanopillar to diminish the optical losses of the metallic plasmonic nanocavity that is formed (Figure 1f).^40–42^ The scanning electron microscopy (SEM) images of a periodic array of pillars are shown in Figure S2 (see also Section S3 and S4). For small twist angles θ, the momentum mismatch between electrons near the *K*_M_ point of MoSe_2_ and holes near the *K*_W_ point of WSe_2_ is about $\frac{\theta }{360}\frac{2\pi }{a}$ , where *a* is the lattice constant of the hexagonal real space lattice of MoSe_2_ and WSe_2_ and the angle θ is in degrees.^19,38,43^ The lattice constants of MoSe_2_ and WSe_2_ are 3.326 and 3.325 Å, respectively.^44^ For a twist angle of 4°, this yields an in-plane momentum mismatch of approximately 0.21 nm^–1^. The momentum of light for a wavelength λ_0_ of, for example, 730 nm in free space is *k*_0_ = 2π/λ_0_ = 0.86 × 10^–2^ nm^–1^, which is more than 1 order of magnitude smaller than the momentum mismatch. However, due to the spatial confinement of the near-field light in the nanocavity, an electric field composed of an angular spectrum of plane waves and evanescent waves exists in the cavity. The evanescent waves possess an imaginary component of the wave vector in the *z* direction (*k*_*z*_) with a maximum magnitude set by the vertical distance separating the gold film and the gold pillar *d*. For *d* = 7.5 nm in the device studied here, this magnitude amounts to 0.84 nm^–1^. This effectively also broadens the in-plane photon wave vector (*k*_in_) and momentum because the magnitude of the overall wave vector still equals the far-field value and the large negative contribution from the evanescent part should be compensated for ( $k=\sqrt{{k_{z}}^{2}+{k_{\mathrm{in}}}^{2}}=n\frac{\omega }{c}$ ).^45^

**Figure 2:**
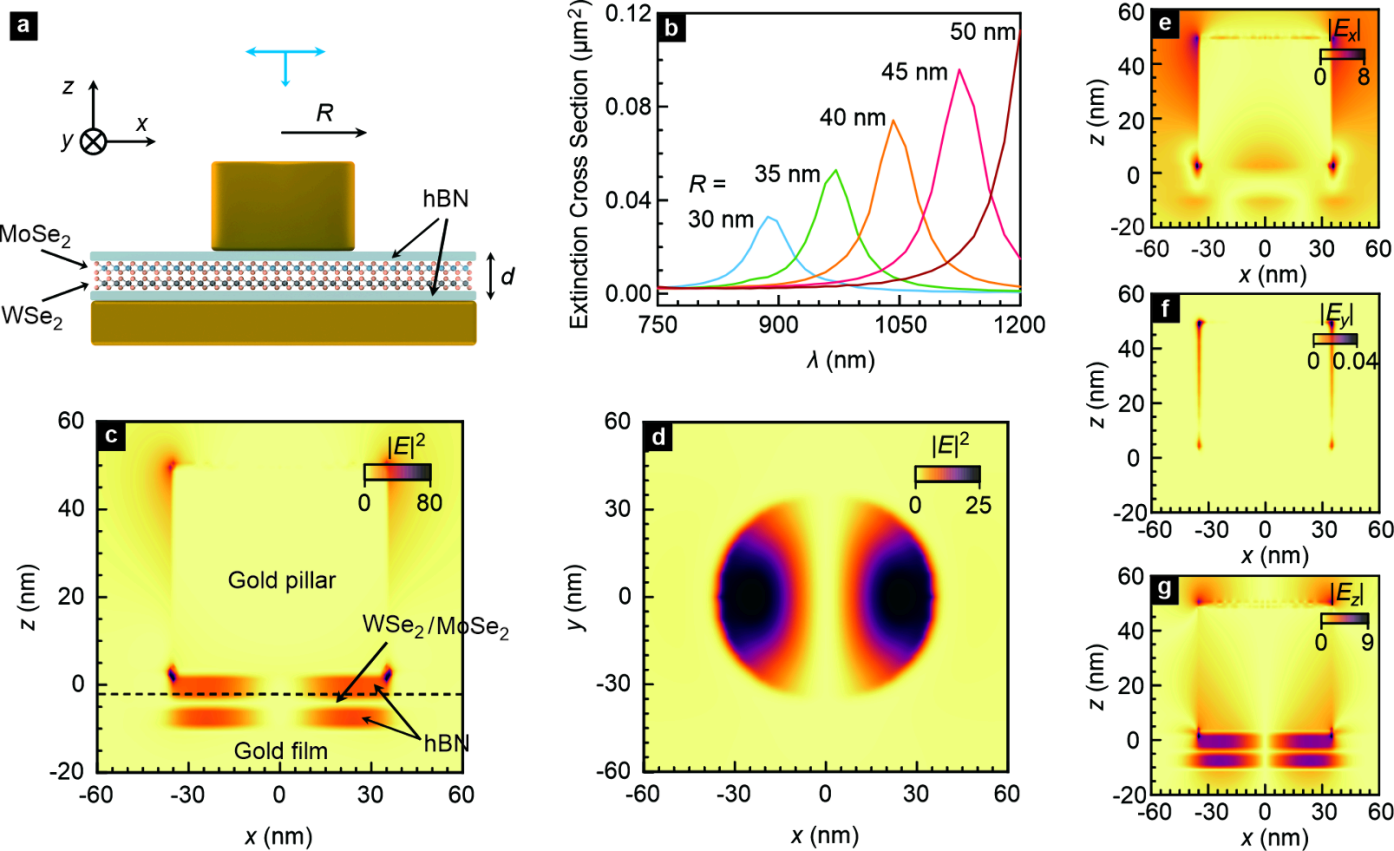
Electric field distribution for a WSe_2_/MoSe_2_ heterostructure embedded in a plasmonic nanocavity. (a) Cross section of a WSe_2_/MoSe_2_ based van der Waals heterostructure incorporated into a plasmonic nanocavity. The radius of the nanopillar on top of the heterostructure is *R*, whereas *d* is the distance between the gold film deposited on the substrate and nanopillar. (b) Simulation results of the extinction cross section of the cavity as a function of the wavelength λ for *R* varying from 30 to 50 nm. (c) Calculated spatial distribution of the electric field intensity |*E*|^2^ in the *x*–*z* plane in the vicinity of the cavity for *y* = 0. For a definition of the coordinate system, see Section S4 and Figure S3. (d) Spatial distribution of |*E*|^2^ in the *x*–*y* plane near the cavity for *z* = −3 nm (black dotted line in panel c). Same as panel c but for the magnitude of each electric field component: |*E*_*x*_| (e), |*E*_*y*_| (f), and |*E*_*z*_| (g). The distribution of these field components in the *x*–*y* plane is shown in Section S6.

The calculated extinction cross section at *d* = 7.5 nm with varying cavity radius (*R*) is plotted in Figure 2b. Here, we show the spatial distribution of the electric field intensity |*E*|^2^ as well as the three different electric field components in the selected device geometry. The calculations are performed for a cavity radius *R* of 35 nm. Figure 2c displays a color rendition of the overall electric field intensity in the *x*–*z* plane for *y* = 0 (see the coordinate system in Figure S3) and 730 nm excitation light. The “hot spot” of the field intensity is in the hBN/WSe_2_/MoSe_2_/hBN spacer region. The *x*–*y* cross section of the field intensity at the interface between MoSe_2_ and the top hBN is shown in Figure 2d. This field intensity in the spacer region stems from a magnetic dipole mode and is primarily located at the boundary of the area covered by the gold nanopillar. Panels e–g are color renditions of the amplitude of the *x*, *y*, and *z* components of the electric field across the (*x*, *z*) plane at *y* = 0. Only the amplitude of *E*_*z*_ is very significant in the spacer region (Figure 2e–g). Because the dipole moment of the interlayer excitons is parallel to the *z* axis (Figure 1c), this field component can couple effectively to the interlayer excitons, and we anticipate a strong local density of optical states (LDOS) in the spacer region.^42,46^ The magnitude of the fast Fourier transform of the complex *E*_*z*_ is included in Section S7 and reveals that this field component indeed has intensity at sufficiently large momenta to overcome the twist angle induced momentum mismatch and assist with indirect electronic transitions between MoSe_2_ and WSe_2_ without phonon participation due to the momentum “broadening” by spatial confinement imposed by the nanocavity.

Not only the momentum content of the ONF but also the strong LDOS enhances the interlayer exciton emission through several additional contributions. The above electric field simulations can be used to calculate the enhancement factor for optical excitation γ_exc_/γ_exc_^0^ = |*E*|^2^/|*E*_0_|^2^, where γ_exc_ and |*E*|^2^ are the excitation rate and field intensity, if the sample is embedded in the cavity.^40,42^ The corresponding quantities in the region without the top nanopillar carry a super- or subscript 0. The calculation results for different *R* can be found in Figure S7a.

Also the spontaneous emission rate γ_sp_ gets boosted. According to Fermi’s golden rule, γ_sp_ of a dipole is given by^42,47,48^

$$\gamma_{\mathrm{sp}}\left(r\right)=\frac{\pi \omega }{3\hbar \epsilon_{0}}{\left|p\right|}^{2}\rho \left(r,\omega \right)$$ 1

where ω is the emission frequency, ϵ_0_ is the permittivity of free space, ***p*** is the transition dipole moment of the emitter, and ρ­(***r***,ω) is the LDOS at frequency ω and emitter position ***r***. By placement of the WSe_2_/MoSe_2_ heterostructure in a cavity, the spontaneous emission rate benefits from the enlarged LDOS through the Purcell effect.^42,47,48^ This effect has been exploited previously for improving the radiation efficiency of excitons.^18,22–25^ The Purcell factor (*F*_P_) can be written as^46,48^

$$F_{P}=\frac{\gamma_{\mathrm{sp}}}{\gamma_{0}}=\frac{3}{4\pi^{2}}{\left(\frac{\lambda }{n}\right)}^{3}\frac{Q}{V_{\mathrm{mode}}}$$ 2

Here, γ_0_ is the spontaneous emission rate of the emitter in free space, *Q* is the quality factor of the cavity, *V*_mode_ is the cavity mode volume, *n* is the refractive index of the spacer medium, and λ is the resonant wavelength. *Q* varies from 11.8 to 14.6 and *V*_mode_ varies from 440 to 2020 nm^3^ with a change of *R* from 35 to 50 nm, as can be seen in Figure S8.

The calculated Purcell factor depends on the position of the emitter underneath the pillar, as shown in Figure 3a. For dipoles oriented along the *z* direction, the Purcell factor is about 9000 near the boundary of the pillar with *R* = 35 nm (Figure 3b). In view of the indirect nature of the interlayer excitons in reciprocal space, we adopt the case of low quantum yield (QY) for which the nonradiative decay γ_nr_ ≫ *F*_P_γ_sp_. The enhancement of the emission quantum yield is then equal to QY/QY^0^ ≈ *F*_P_/*F*_P_^0^, where *F*_P_ and *F*_P_^0^ are the Purcell factors with and without the gold nanopillar above the top hBN.^40,49^ The dependence of the quantum yield enhancement on *R* is illustrated in Figure S7b.

**Figure 3:**
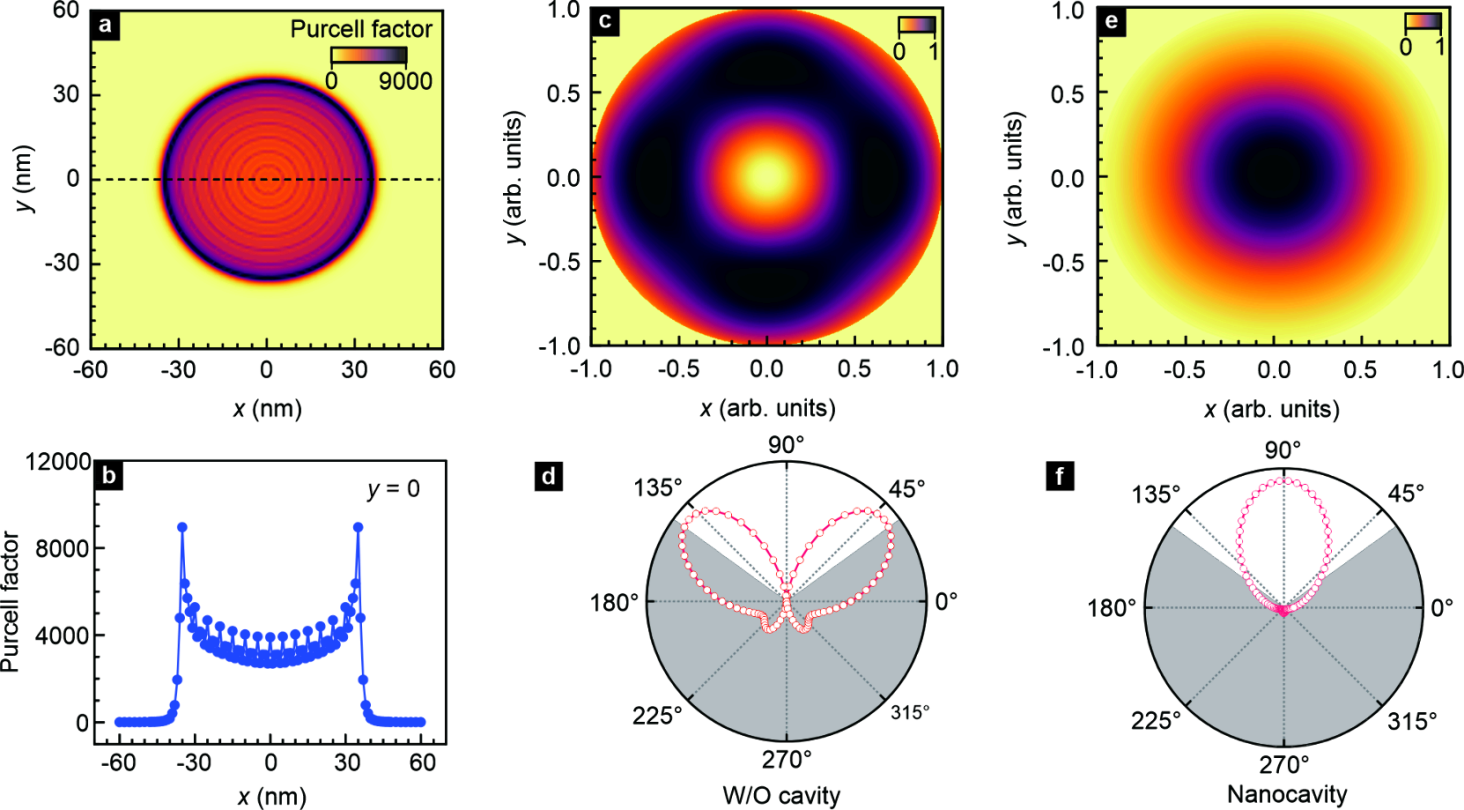
Emission efficiency enhancement and radiation pattern. (a) Calculated spatial distribution of the Purcell factor in the *x*–*y* plane at the position of the WSe_2_/MoSe_2_ heterostructure inside the nanocavity formed by the gold substrate film and gold pillar. (b) Purcell factor along the black dashed line in panel a. (c) Spatial distribution of the normalized far-field radiation power of hBN/WSe_2_/MoSe_2_/hBN heterostructure placed on top of a gold film. (d) Polar diagram for the far-field radiation shown in panel c. The gray shaded area corresponds to the region from which no signal can be collected with the chosen objective lens with a numerical aperture of 0.81. (e) Same as panel c but for the case where the heterostructure is embedded in a gold cavity. The out-of-plane dipole is put at the boundary of the gold nanopillar. (f) Polar diagram for the far-field radiation shown in panel e. The emission wavelength of the dipole is set at 880 nm.

Finally, also the collection efficiency for the emitted radiation from interlayer excitons is improved in the selected geometry. The simulated distribution of the power of the far-field radiation from an hBN-encapsulated WSe_2_/MoSe_2_ heterostructure placed on a gold film or between a gold film and a gold pillar for an objective with a numerical aperture of 0.81 is plotted in Figure 3c,e. In the absence of the pillar, the emission pattern has the shape of a butterfly (Figure 3d) and the collection efficiency (η) from the emission from *z*-oriented dipoles is estimated to be only 38%. For a nanopillar with *R* = 35 nm on top of the van der Waals heterostructure, the spatial distribution of the far-field radiation power is modified into a single upward lobe, as seen in Figure 3f,^42^ and the collection efficiency for this case is calculated to be 73% instead (Figure 3e,f). Hence, the enhancement factor η/η^0^, with η and η^0^ the collection efficiency with and without plasmonic cavity,^40,42^ is as large as 1.92. The expected variation of this enhancement as a function of *R* is shown in Figure S7c.

The emission enhancement for the cavity geometry is confirmed by the experimental data. Figure 4a illustrates the photoluminescence (PL) spectrum obtained in reflection microscopy on a misaligned WSe_2_/MoSe_2_ heterostructure when the heterostructure is not embedded in a cavity. The emission signal is very weak and exhibits a maximum near 870 nm. To confirm that this emission stems from interlayer excitons, photoluminescence excitation (PLE) measurements are performed. As the excitation wavelength is tuned, the emission at 870 nm intensifies at two resonant wavelengths, as seen in Figure 4b. These are identified more precisely by taking the integral of the emission intensity across the full spectral range and plotting the result as a function of the excitation wavelength (Figure 4c). Peaks appear at 729 nm as well as 764 nm, the wavelengths where light is absorbed in WSe_2_ and MoSe_2_ monolayers due to A excitons or trions (see the PL in Section S10). We conclude that the emission at 870 nm involves both WSe_2_ and MoSe_2_ and originates from interlayer excitons that form at their heterointerface.^25^

**Figure 4:**
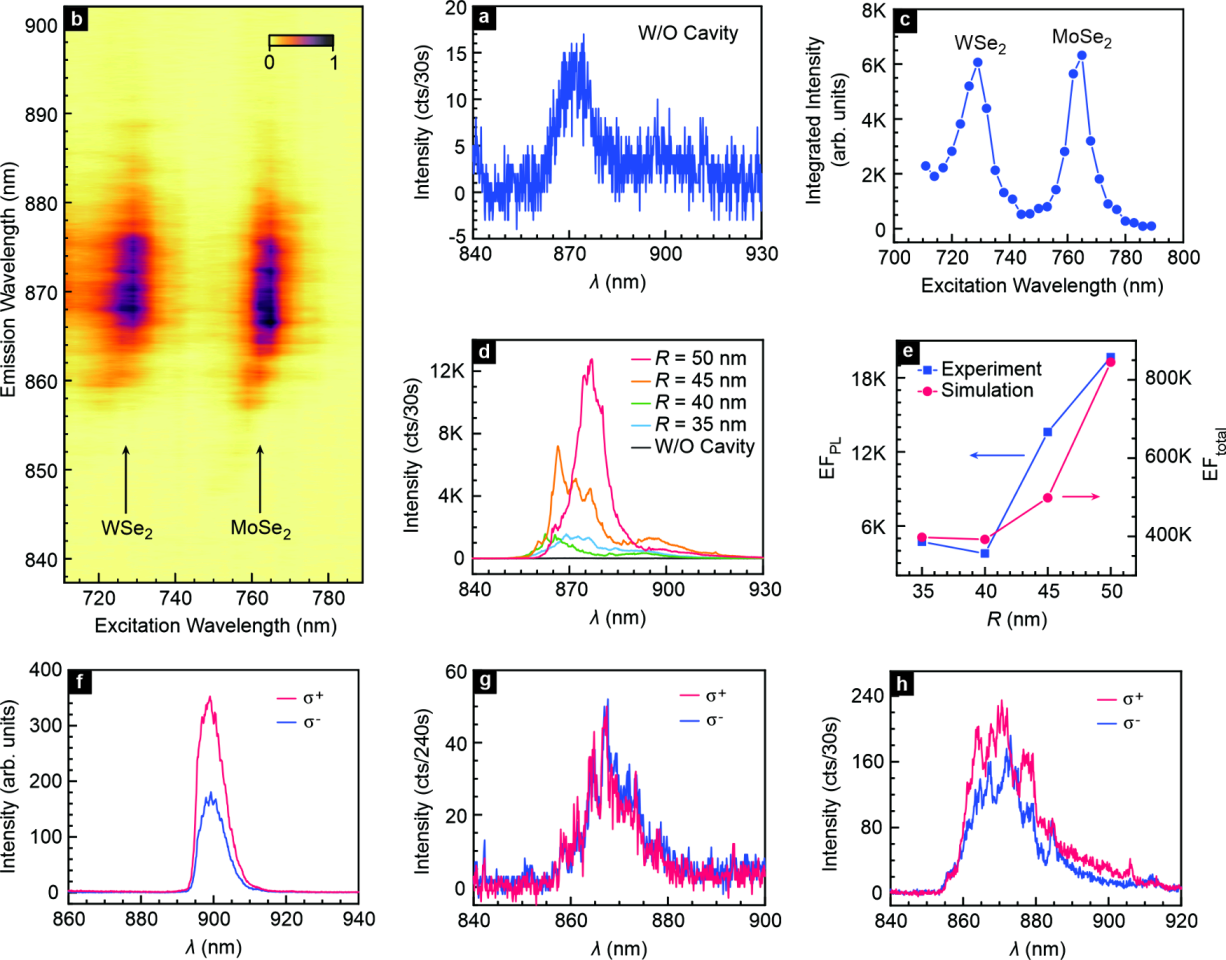
Experimental evidence for enhanced emission from momentum indirect excitons in a twisted WSe_2_/MoSe_2_ heterostructure. (a) PL spectrum recorded for an excitation wavelength of 730 nm laser when the hBN-encapsulated heterostructure is just placed on the gold substrate film. (b) False color image of the intensity of the interlayer exciton emission as a function of the excitation and emission wavelengths for the sample in panel a. (c) Integrated intensity of the interlayer exciton emission as a function of the excitation wavelength in panel b. (d) Comparison of the PL spectrum recorded on a twisted WSe_2_/MoSe_2_ heterostructure when the heterostructure is just placed on top of a gold film (without cavity, w/o cavity) or embedded in a cavity with different radii. The incident laser power is 1 μW and has a wavelength of 730 nm. (e) PL enhancement factor EF_PL_ as extracted from the experiment (blue squares) and the calculated total enhancement factor EF_total_ (red circles) as a function of the radius *R* of the gold pillar. (f) Circular polarization-resolved PL spectra recorded on an antialigned (60° twist angle) WSe_2_/MoSe_2_ heterostructure without cavity. (g and h) Circular polarization-resolved PL spectra recorded on a twisted WSe_2_/MoSe_2_ heterostructure placed on top of the gold substrate film (g) and located within a cavity (h). The data were recorded for *R* = 35 nm. The 730 nm laser light with a power of 1 μW incident on the sample was circularly polarized (σ^+^ polarization). All measurements were recorded at 2 K.

We now turn our attention to the impact of embedding the twisted heterostructure into a plasmonic nanocavity to boost the emission from the momentum indirect interlayer excitons. The evolution of the experimental PL spectrum recorded on the WSe_2_/MoSe_2_ heterostructure covered with gold pillars for different radii (35, 40, 45, and 50 nm) is shown in Figure 4d. Even for the smallest radii, the enhancement is significant compared to the case where no cavity is formed, but it becomes truly gigantic for larger radii. Besides, the moiré potential may compress the exciton wave function and increase the electron–hole overlap integral, resulting in a higher LDOS and oscillator strength for localized excitons. Figure 4d shows that the broad emission spectrum of the interlayer excitons features some maxima. These can be attributed to the presence of interlayer excitons trapped in the moiré potential.^25^ These features too get enhanced when the van der Waals heterostructure is embedded in a cavity. To quantify the boost in the PL intensity, we introduce the PL enhancement factor (EF_PL_) defined as^40,42^

$${\mathrm{EF}}_{\mathrm{PL}}=\frac{I_{\mathrm{cavity}}}{A_{\mathrm{cavity}}}{\left(\frac{I_{W/O}}{A_{\mathrm{laser}}}\right)}^{-1}$$ 3

Here, *I*_cavity_ and *I*_W/O_ are the PL intensity integrated over the recorded spectral range when the heterostructure is embedded inside a cavity (*I*_cavity_) or just placed on top of the gold film (*I*_W/O_). Both intensities are weighted by the area the emission stems from. When the hBN-encapsulated heterostructure is just placed on the gold film this area is equal to the laser spot size, *A*_laser_. For the heterostructure with photon confinement, it is the cavity area (*A*_cavity_ = *n*π*R* ^2^) under the laser spot. For *R* = 50 nm, EF_PL_ is as large as 2 × 10^4^. Because most of the emission originates from the boundary of the gold pillar, the actual area from which PL is gathered is smaller and, hence, the calculated EF_PL_ is an underestimate. The experimental EF_PL_ drops a lot for heterostructures with larger twist angles because of insufficient near-field momentum (Section S11).

We also consider the calculated total enhancement factor, EF_total_. It combines the excitation enhancement (γ_exc_/γ_exc_^0^), the quantum yield enhancement (QY/QY^0^), as well as the collection efficiency enhancement (η/η^0^) and is just equal to the product of these three:^40,42,48^

$${\mathrm{EF}}_{\mathrm{total}}=\frac{\gamma_{\mathrm{exc}}}{\gamma_{\mathrm{exc}}^{0}}\frac{\mathrm{QY}}{{\mathrm{QY}}^{0}}\frac{\eta }{\eta^{0}}$$ 4

Its calculated value as a function of *R* is included in Figure 4e (right vertical axis). The simulated EF_total_ is much larger than the experimental EF_PL_ because in the calculation the highest γ_exc_/γ_exc_^0^, QY/QY^0^, and η/η^0^ are utilized.

The resonant wavelength of the plasmonic cavity exhibits a red shift by more than 300 nm when its radius *R* is tuned from 30 to 50 nm, as shown in Figure 2b. In the experiment, we can address cavities starting from 35 nm due to processing limitations, but we find that this smallest cavity is not optimal, neither in experiment nor in calculations. Due to the interplay of the various contributions to the enhancement factor, *R* = 50 nm yields the highest enhancement. For instance, the Purcell factor exhibits an additional maximum at the emission wavelength for *R* = 50 nm. Further details on the origin of this peak can be found in Section S12.

The drastic enhancement of the interlayer exciton emission in the chosen sample configuration is attributed to two factors: the momentum content in the ONF, which assists indirect electronic transitions, in conjunction with the influence of plasmons. The importance of the former can be experimentally confirmed by studying the circular polarization dependence of the PL. For aligned or antialigned heterostructures where there is no mismatch in momentum, phonon scattering is not required to establish interlayer excitons and valley polarization can build up upon excitation with circularly polarized light. As a result, the emission exhibits a strong polarization dependence, as illustrated in Figure 4f. Because the device is excited with σ^+^ polarization, σ^+^ emission dominates. In contrast, for a twisted heterostructure not embedded in a cavity the emission strength is essentially identical for both circular polarization directions (Figure 4g). The momentum mismatch calls for phonon assisted scattering and valley polarization is lost. If the heterostructure is embedded in a cavity, the polarization dependence is partially recovered (Figure 4h), because phonon scattering is no longer required during interlayer exciton formation and recombination. For heterostructures with a larger twist angle, the larger momentum mismatch becomes harder to compensate for with the momentum content of the ONF, and the enhancement factor is expected to drop substantially. This is corroborated by an experiment on a sample with a 14° twist shown in Section S11. For the sake of completeness, we note that the enhancement of the radiative recombination by the Purcell effect may also play a role in the increase of circular polarization because of the reduced interaction time for depolarization processes.

The twist angle has long been identified as a powerful knob to tune the optical properties of van der Waals heterostructures based on TMDCs. A drawback has, however, been the low intensity of the optical emission due to the twist-angle-induced momentum mismatch that converts these systems into indirect-gap materials. Here we have demonstrated that it is possible to overcome this weak light–matter interaction and fully benefit from the properties of the twist-angle-engineered interlayer excitons by placing the heterostructure inside a plasmonic nanocavity. Both the radius and thickness of the cavity are key design parameters to adjust the Purcell effect and adapt the momentum of the near-field light to the electronic momentum mismatch in order to outwit the indirect nature of the electronic gap. A boost in the optical emission of momentum indirect interlayer excitons of up to 4 orders of magnitude was achieved in this way.

## Supplementary Material



## Notes

Details of the experimental methods, determination of the twist angle, SEM images of the fabricated nanocavities, sample configurations and definition of the coordinate system, estimate of the in-plane momenta available in the ONF, absorption and scattering cross section, in-plane dependence of the electric field components, radius-dependent enhancement of the excitation, quantum yield, and collection efficiency, *Q* and *V*_mode_ of the plasmonic nanocavity, PL spectrum recorded on a WSe_2_ and MoSe_2_ monolayer, PL and EF_PL_ for a WSe_2_/MoSe_2_ heterostructure with a 14° twist angle, and discussion of the dependence of EF_PL_ on the cavity size (PDF)
