# Supplementary material for: Boosting the Emission of Momentum Indirect Interlayer Excitons by an Optical Near Field in Misaligned 2D Heterostructures
Source: Nano Lett. 2025 Oct 7;25(41):14800–7. doi: 10.1021/acs.nanolett.5c02703 (PMC12532282; doi:10.1021/acs.nanolett.5c02703)
Supplement: Supplementary file 1 [file nl5c02703_si_001.pdf]

Supplementary Information

Boosting the Emission of Momentum Indirect Interlayer Excitons by an Optical Near Field in Misaligned 2D Heterostructures

Qixing Wang[1,2], Takashi Taniguchi[3], Kenji Watanabe[4] and Jurgen H. Smet[*2]*

[1]Department of Physics, College of Physical Science and Technology, Xiamen University, Xiamen 361005, China; Jiujiang Research Institute, Xiamen University, Jiujiang 332000, China

[2]Max Planck Institute for Solid State Research, Stuttgart D-70569, Germany

[3]Research Center for Materials Nanoarchitectonics, National Institute for Materials Science, Tsukuba, Japan

[4]Research Center for Electronic and Optical Materials, National Institute for Materials Science, Tsukuba, Japan

Corresponding author: Jurgen H. Smet (Email: j.smet@fkf.mpg.de, Tel: +49 711 689-5244)

Supplementary sections

S1. Experimental methods

S2. Determination of the twist angle

S3. SEM images of the fabricated nanocavities

S4. Sample configurations and definition of the coordinate system

S5. Absorption and scattering cross section

S6. The in-plane dependence of the electric field components

S7. Estimate of the in-plane momenta available in the optical near field

S8. Radius dependent enhancement of the excitation, quantum yield and collection efficiency

S9. Q and V_{mode} of the plasmonic nanocavity
S10. PL spectrum recorded on a WSe_2 and $MoSe_2$ monolayer
S11. PL and EF_{PL} for a WSe_2/$MoSe_2$ heterostructure with a 14° twist angle
S12. Discussion of the dependence of EF_{PL} on the cavity size

S1. Experimental methods

Simulations. Maxwell's equations were solved using the Finite Difference Time Domain (FDTD) method. The absorption, scattering, and extinction cross sections were obtained by illuminating the structure with a Total-Field Scattered-Field plane wave source (TFSF). The electric field distribution was calculated by using a plane wave polarized along the *x*-axis and propagating perpendicular to the substrate surface. Interlayer excitons were modelled as monochromatic point dipoles oscillating along the z-axis at an emission wavelength of 880 nm. The Purcell factor was simulated by placing a point dipole at the heterointerface between the $MoSe_2$ monolayer and the top hBN underneath the gold pillar. The spatial distribution of the Purcell factor was calculated by varying the position of the dipole emitter. The spatial distribution of the far field radiation power was simulated by placing a dipole that oscillates along the *z*-axis (λ = 880 nm) at the $MoSe_2$/hBN heterointerface within the cavity. The collection efficiency was evaluated by integrating the power of the emitted radiation collected by an objective lens with a numerical aperture NA = 0.81 as in experiment and dividing the result with the total emitted power across all angles.

Sample preparation. A titanium/gold film (5 nm/50 nm) was deposited on top of a Si substrate covered with a 300 nm thick thermal SiO_2 using a thermal evaporator. $MoSe_2$ and WSe_2 monolayers as well as hBN multilayer flakes were exfoliated from their bulk crystals on polydimethylsiloxane (PDMS) stamps.[1-4] Subsequently, a heterostructure composed of a bottom hBN layer, a WSe_2 monolayer, a $MoSe_2$ monolayer and a top hBN layer was fabricated using van der Waals stacking with a home-built transfer stage. The full layer sequence was then released on top of the substrate covered with the titanium/gold film. The sample was annealed in a forming gas atmosphere (W10 forming gas with 10% H_2) at a pressure of 150 mbar and temperature of 200 °C for 2 hours. To determine the relative orientation of the two optically active layers, i.e. the $MoSe_2$ and WSe_2 monolayers, the polarization dependence of the second-harmonic generation

response of the two layers was studied.[5, 6] The thicknesses of the top and bottom hBN layers were measured with atomic force microscope (AFM). Finally, gold nanopillars with varying radii were patterned on top of the van der Waals heterostructure with the help of electron beam lithography (EBL) and lift-off. To this end, a 0.5 nm Cr layer followed by a 50 nm thick gold film were thermally evaporated in order to form a plasmonic nanocavity. Arrays of cavities were fabricated with a distance of nominally 200 nm between two adjacent pillars in both orthogonal directions. The shape of the cavities was verified and characterized with scanning electron microscopy (SEM).

Optical measurements. Micro-photoluminescence (μ-PL) measurements were carried out with a home-built confocal microscope based on an objective with a numerical aperture NA=0.81. Samples were mounted on a holder for a cryostat with a variable temperature insert that can be operated down to a base temperature of 2 K. The light propagates in free space and the samples are studied in a reflection geometry. The optical excitation stems from a tunable continuous wave (CW) Ti:sapphire laser (Coherent). The pump power was set at 1μW unless otherwise specified. The emitted photoluminescence (PL) signal was detected with a liquid nitrogen-cooled charge coupled device (SynapsePlus BIDD CCD) installed on a monochromator (iHR-320) with a 600 grooves/mm grating. Circular polarization-resolved μ-PL spectra were recorded using a linear polarizer and a quarter-wave (λ/4) plate in both the excitation and collection path.[7] For the calculation of the PL enhancement factor (EF_{PL}), the geometrical area of the cavity is taken as $A_{cavity} = n\pi R^2$, where n is the number of pillars illuminated by the laser with a diameter of the laser spot of 1 μm.

S2. Determination of the twist angle

With some effort it is possible to experimentally determine the twist angle of a $WSe_2/MoSe_2$ van der Waals heterostructure using second harmonic generation (SHG). Here, we show exemplary SHG data recorded on one of our heterostructures. Figure S1a displays the SHG intensity integrated over the measured spectral range (see Fig. S1b) as a function of the rotation angle θ of the sample stage for the $MoSe_2$ (black squares) and WSe_2 (red circles) monolayer regions away from the overlap area. The excitation wavelength was equal to 780 nm (fs) and the incident laser power was 4 mW. Both data sets have been normalized to their highest integrated intensity for

ease of comparison. The data points are fitted with the formula $I = A + I_0 cos^2(3(\theta - \theta_0))$. Here I is the integrated SHG intensity. A, I_0 and θ_0 are fit parameters. More specifically, θ_0 is the angle between the horizontal direction of the sample stage and the armchair direction of the measured 2D layer.[5, 6] The difference between the θ_0 values extracted for the WSe_2 and $MoSe_2$ monolayer through fitting is the sought-for twist angle between the layers and is in the example shown equal to either 4° or 56°. Panel b shows the spectrum of the SHG signal recorded on the two monolayers separately as well as on the heterostructure area for one specific sample stage angle. These measurements were performed for the same excitation wavelength, but with a laser power of 1 mW only. The SHG intensity is larger for the heterostructure region than the sum of the signals of the two monolayers. This is due to constructive interference and allows us to conclude that the twist angle is 4° and not 56° in this sample (Figure S1b).[5, 8]

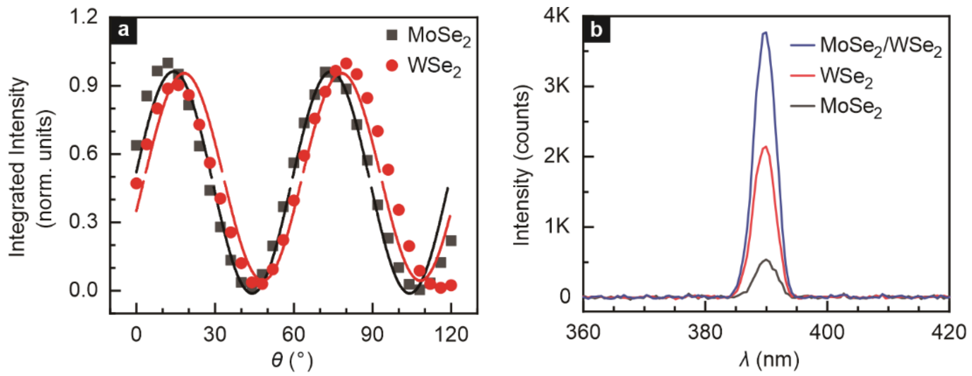

Figure S1. Second-harmonic generation (SHG) data recorded on a $WSe_2/MoSe_2$ van der Waals heterostructure. (a) Normalized integrated intensity as a function of the rotation angle θ of the sample stage recorded on the $MoSe_2$ (black squares) and WSe_2 (red circles) monolayer in regions where these layers do not overlap. Lines are least square fits to the data with the formula $I = A + I_0 cos^2(3(\theta - \theta_0))$. (b) SHG spectrum covering the wavelength range λ from 380 to 400 nm for the $MoSe_2$ monolayer (black), the WSe_2 monolayer (red) and the $MoSe_2/WSe_2$ overlap area (blue).

S3. SEM images of the fabricated nanocavities

Figure S2a shows SEM images of the array of plasmonic nanocavities fabricated by electron beam lithography, thermal evaporation and lift-off. It consists of nanopillars with a diameter *D* of about 70 nm. The periodicity of the array amounts to 270 nm, so that the space *L* separating two adjacent pillars equals 200 nm. This distance was selected in order to reduce the optical coupling. Both *D* and *L* are illustrated in Figure S2b.

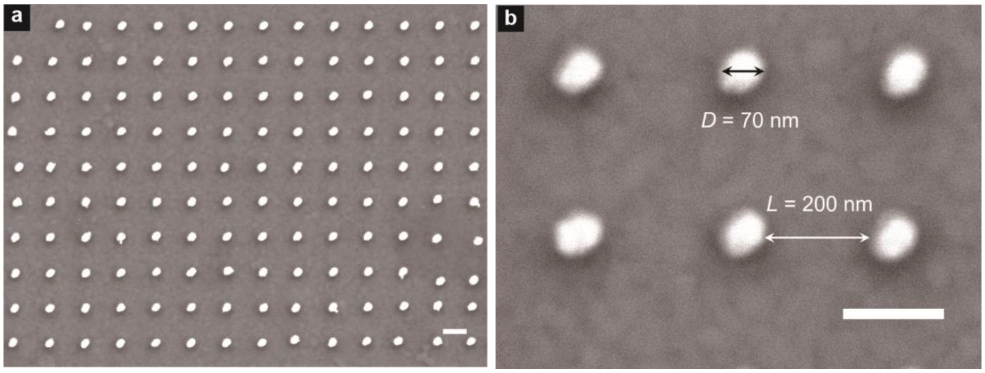

Figure S2. SEM images of the array of plasmonic nanocavities. (a) SEM image. The white scale bar corresponds to 200 nm. (b) SEM image of the same sample but for a larger magnification. The scale bar also corresponds to 200 nm.

S4. Sample configuration and definition of the coordinate system

Figure S3a shows a side view (not drawn to scale) of the sample configuration for simulating the absorption cross section, the scattering cross section and the electric field distribution. The sample is composed of a $MoSe_2$- and WSe_2-monolayer sandwiched between two hBN mulitlayers and placed on top of a substrate covered with a 5 nm thick adhesion layer and a 50 nm thick gold film. The thicknesses of the top and bottom hBN layers are equal to 3.5 nm and 3.0 nm, respectively. The thickness of the $MoSe_2$ and WSe_2 layers are assumed to be 0.5 nm each. The $MoSe_2$ is located on top of the WSe_2. The nanopillar with radius *R* is made up of 0.5 nm of Cr and 50 nm of Au. The origin of the coordinate system is included in Figure S3a (side view) as well as the top view in Figure S3b. The origin (x = 0, y = 0, z = 0) is located at the center of the bottom surface of the gold nanopillar. The z-axis is normal to the surface of the gold substrate film and points upward. The *x*- and *y*-axis are parallel to the plane formed by the gold film. This coordinate system is used for simulations with the Finite Difference Time Domain (FDTD) method (section S5). The

simulations are performed for a plane wave that oscillates along the *x*-axis and propagates in the -*z*-direction (blue arrow in Figure S3a). In simulations addressing the Purcell factor and radiation pattern, vertical dipoles are put at the heterointerface between the $MoSe_2$ monolayer and the top hBN cap layer. Such a vertical dipole (red double arrow) is schematically drawn at the boundary of the gold nanopillar in Figure S3a.

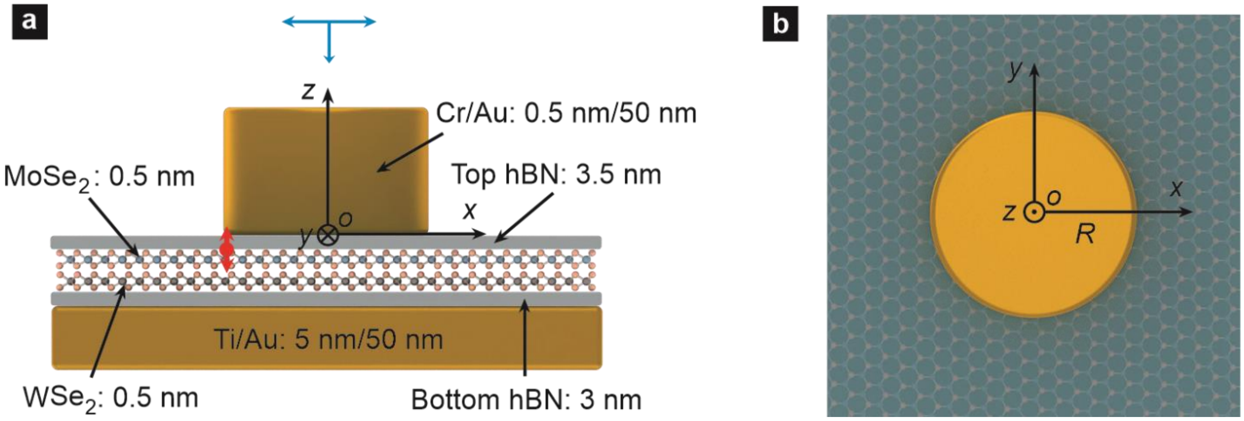

Figure S3. Sample configuration and coordinate system used in the simulations. (a) Sample configuration for simulating the absorption cross section, the scattering cross section and the distribution of the electric field. These simulations are carried out for a plane wave that oscillates along the *x*-axis and propagates along the -*z*-direction (blue arrow). The vertical dipole used in the simulation of the Purcell factor is included in the side view on the left boundary of the nanopillar at the $MoSe_2$/hBN heterointerface as a red double-sided arrow. (b) Top view of the device configuration.

S5. Absorption and scattering cross section

The absorption and scattering cross sections were calculated by solving Maxwell's equations using the Finite Difference Time Domain (FDTD) method for the sample configuration explained in section S4 and shown in Figure S3. The index of refraction n of WSe_2 and $MoSe_2$ were set equal to 4 and 5, respectively (https://refractiveindex.info/). For multilayer hBN the index of refraction is anisotropic. Here, we use in-plane refractive indices $n_x = n_y = 1.65$ and an out-of-plane refractive index n_z equal to 2.13 (https://refractiveindex.info/). A Total-Field Scattered-Field (TFSF) plane wave source propagating along the -*z*-direction was used to illuminate the structure and calculate

the absorption and scattering cross-sections. The outcome of the simulations for cavities with four different radii (30 nm to 50 nm) is shown in Figure S4 for an excitation wavelength ranging from 750 nm to 1200 nm. The resonant wavelength for the cavities is 890 nm, 968 nm, 1044 nm and 1127 nm for R = 30 nm, 35 nm, 40 nm and 45 nm, respectively. The extinction cross section equals the sum of the absorption and the scattering cross section.

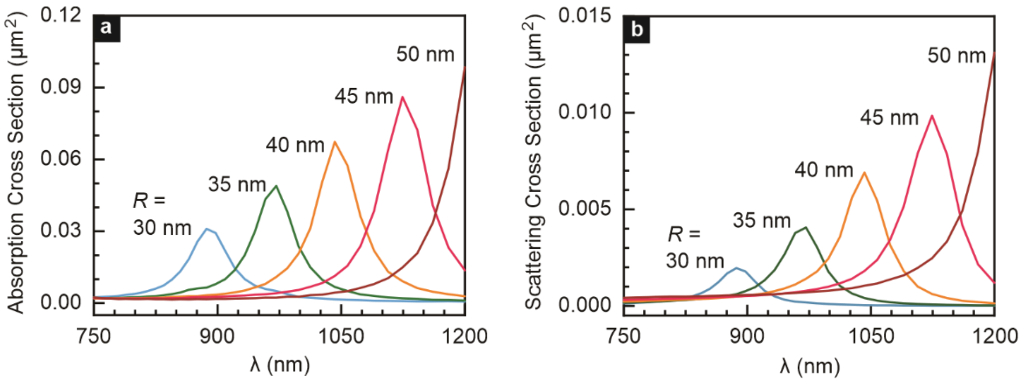

Figure S4. Calculations of the absorption and scattering cross sections for nanocavities of different radii. (a) Absorption cross section. (b) Scattering cross section.

S6. The in-plane dependence of the electric field components

Figure S5 displays the in-plane dependence of the strength of the three electric field components $|E_x|$, $|E_y|$, and $|E_z|$ at the interface of the top hBN layer and the $MoSe_2$ monolayer for the sample configuration shown in Figure S3a. The sample is excited with a plane wave with a wavelength of 730 nm propagating along the -z direction as described in section S4. The graphs were obtained with the help of frequency domain field profile monitors. It is apparent that the $|E_z|$ component dominates the overall electric field strength in the region where the twisted $MoSe_2/WSe_2$ heterostructure is capped by the gold pillar.

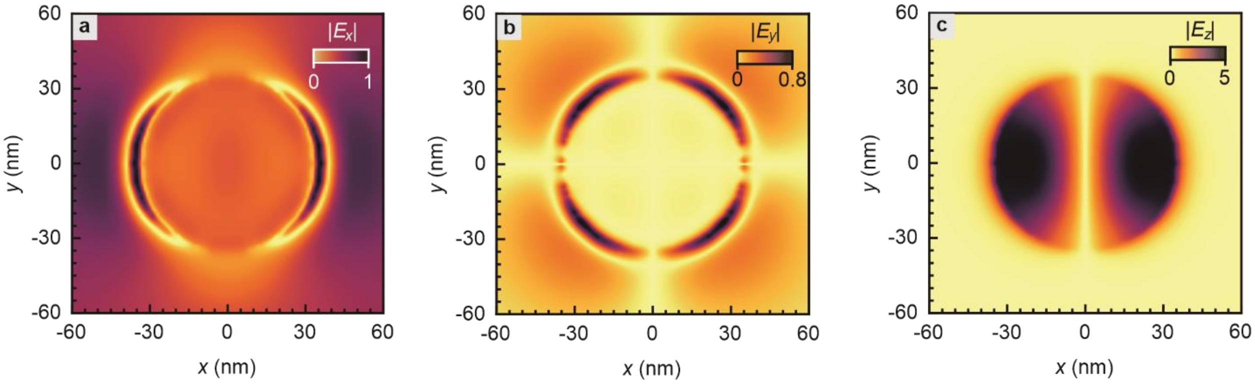

Figure S5. The in-plane distribution of the strength of the electric field components for a plane wave source with 730 nm wavelength. (a) In-plane dependence of $|E_x|$ at the heterointerface between the top hBN and the $MoSe_2$-monolayer, (b) Same as (a) but for $|E_y|$. (c) Same as (a) but for $|E_z|$.

S7. Estimate of the in-plane momenta available in the optical near field

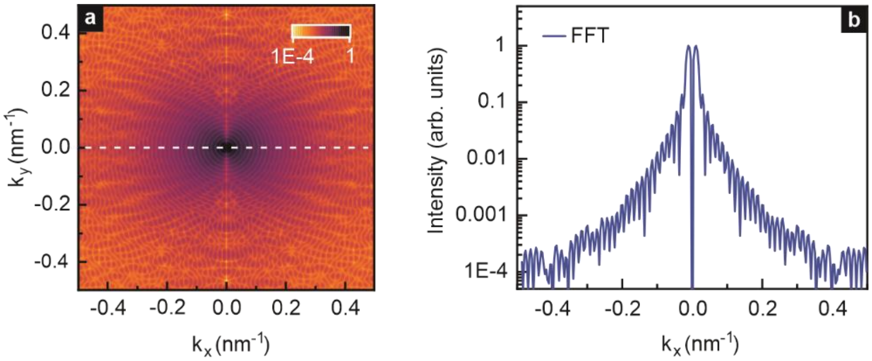

Figure S6. Fast Fourier transformation (FFT) of the complex E_z. (a) The magnitude of the Fast Fourier Transform of the spatial distribution of the complex E_z in Figure S5c. The magnitude is plotted on a logarithmic scale. (b) Line trace along the white dashed in panel (a).

The emission of momentum indirect interlayer excitons exhibits a radiative pattern that corresponds to that of an out-of-plane dipole emitter.[3] As discussed in the main text, the E_z component of the optical near field can couple effectively to such dipoles. Its magnitude was previously calculated and shown in Figure S5c. In order to demonstrate that this field component possesses sufficient momentum to allow for indirect electronic transitions, the magnitude of the

Fast Fourier Transformation (FFT) of the spatial distribution of the complex E_z is plotted in Figure S6a. Figure S6b shows a line trace along the white dotted line in Figure S6a. Even for in-plane wave vectors with a magnitude of 0.5 nm^{-1} there is still sufficient strength of the E_z component and hence the near field supports indirect electronic transitions in the twisted $MoSe_2/WSe_2$ heterostructure without the need for phonon momentum.

S8. Radius dependent enhancement of the excitation, quantum yield and collection efficiency

We normalize the electric field intensity at the $hBN/MoSe_2$ interface for samples with and without coupling to a nanocavity. The resulting quantity is defined as the enhancement factor of optical excitation $\gamma_{exc}/\gamma_{exc}^0 = |E|^2/|E_0|^2$. Figure S7a presents this enhancement factor as a function of the radius R of the nanocavity at the position of the highest electric field intensity encountered in the sample. The enhancement can reach values as high as 150 for R = 50 nm. The quantum yield enhancement factor is defined as $\mathrm{QY}/\mathrm{QY}^0 \approx F_P/F_P^0$, where F_P and F_P^0 are the Purcell factors of the sample with and without coupling to the nanocavity.[9, 10] The highest Purcell factor is achieved for a dipole at the boundary of the nanopillar. Therefore, we calculate the quantum yield enhancement factor at this spot as a function of R. The results are shown in Figure S7b. The enhancement grows with increasing radius R. The collection efficiency η was calculated by integrating the emitted power of a dipole oscillating at a wavelength of 880 nm collected by an objective lens with a numerical aperture NA = 0.81 as in experiment and dividing the result with the total emitted power across all angles. The collection efficiency enhancement factor η/η^0 as a function of R for the emission from a dipole located at the boundary of the nanopillar is depicted in Figure S7c. The collection efficiency decreases with increasing radius.

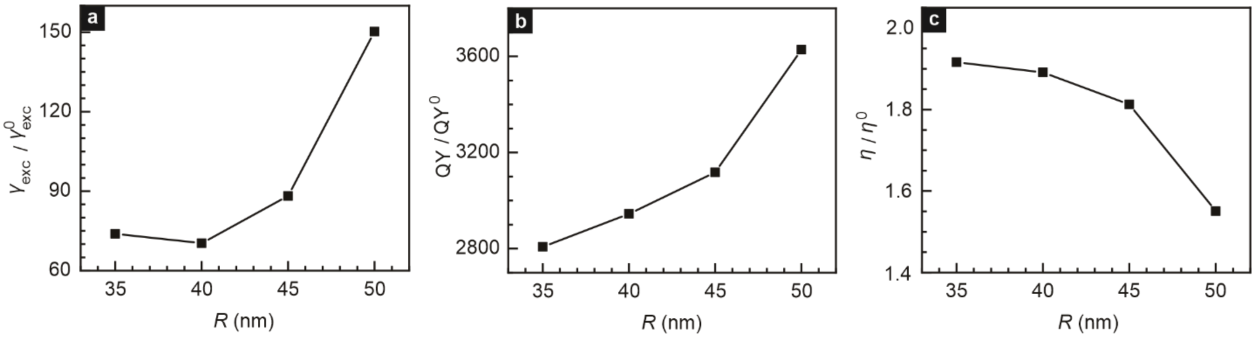

Figure S7. Dependence of the excitation, quantum yield and collection efficiency enhancement on the radius *R* of the nanocavity. (a) The excitation enhancement as a function of *R*. (b) The quantum yield enhancement as a function of *R*. (c) The collection efficiency enhancement as a function of *R*.

S9. Q and V_{mode} of the plasmonic nanocavity

Figure S8a shows the simulated quality factor Q as a function of nanopillar radius R of the designed plasmonic nanocavity. With the increase of R from 35 nm to 50 nm, the Q increases gradually from 11.8 to 14.6. The mode volume V_{mode} of the designed cavity varies from 440 nm^3 to 2020 nm^3 with the change of R as shown in Figure S8b.

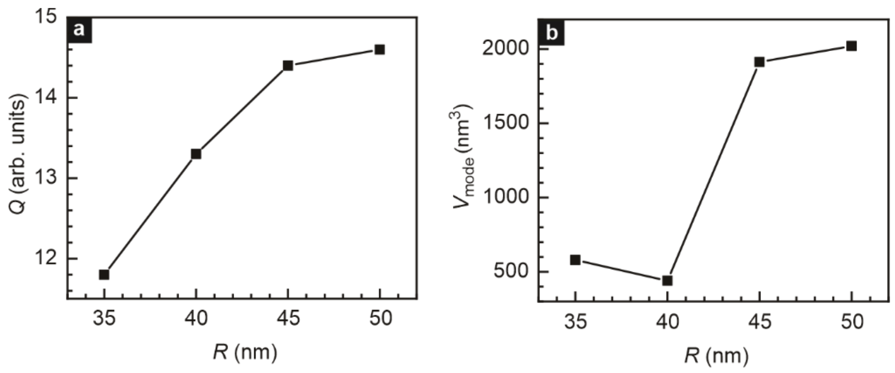

Figure S8. Q and V_{mode} of the plasmonic nanocavity as a function of R. (a) The quality factor Q as a function of R. (b) The mode volume V_{mode} as a function of R.

S10. PL spectrum recorded on a WSe_2 and $MoSe_2$ monolayer

Figure S9a displays the PL spectra of WSe_2 and $MoSe_2$ monolayers exfoliated on SiO_2/Si substrates and illuminated with a 633 nm laser. The PL peak from the A-excitons of a WSe_2 monolayer is centered at 735 nm. The peaks from the A excitons and trions of a $MoSe_2$ monolayer are located at 749 nm and 762 nm, respectively. These results are consistent with previous reports.[11, 12] After stacking WSe_2 and $MoSe_2$ monolayers together, interlayer excitons form upon

illumination in the overlap area (Figure S9b). The absorption of A excitons or trions from WSe_2 and $MoSe_2$ monolayers both facilitate the formation of interlayer excitons. Hence, two resonance peaks appear close to the position of the WSe_2 and $MoSe_2$ A excitons or trions in the PLE spectrum showing the interlayer excitons in a $MoSe_2/WSe_2$ heterostructure (Figure 4b in the main text).[13]

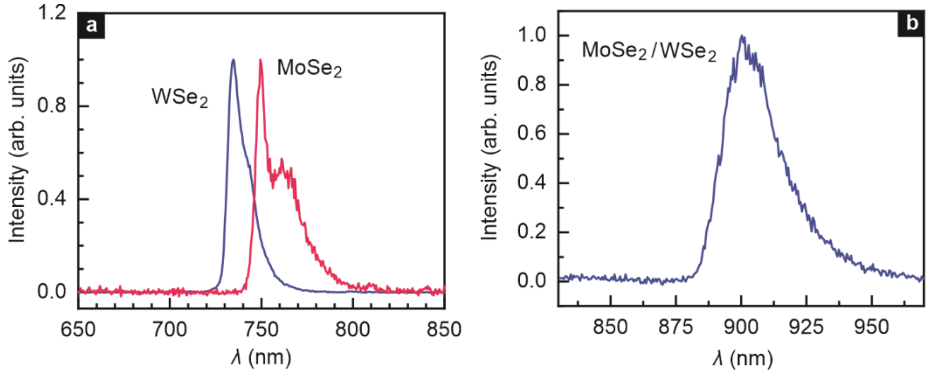

Figure S9. PL Spectra recorded on a WSe_2 and $MoSe_2$ monolayer. (a) PL spectrum recorded on a WSe_2 monolayer (blue) and a $MoSe_2$ (red) monolayer. Data were taken at a temperature of 2 K. The intensity has been normalized. (b) Same as (a) but for the $MoSe_2/WSe_2$ heterostructure region.

S11. PL and EF_{PL} for a $WSe_2/MoSe_2$ heterostructure with a 14° twist angle

Experiments were also performed on a sample with a larger twist angle of 14°. The outcome for cavities with radii varying from 45 to 60 nm is shown in Figure S10a. A clear enhancement of the PL intensity is still observed when the van der Waals heterostructure is incorporated in a cavity, however the enhancement factor EF_{PL} has dropped substantially as seen in Figure S10b. It varies from 32 to 165 as the cavity radius is enlarged. This should be compared with the values in Figure 4e that are at least two orders of magnitude larger. So indeed, for devices with larger twist angles the PL enhancement drops substantially as it is no longer possible to compensate for the larger momentum mismatch with the momentum of the optical near-field.

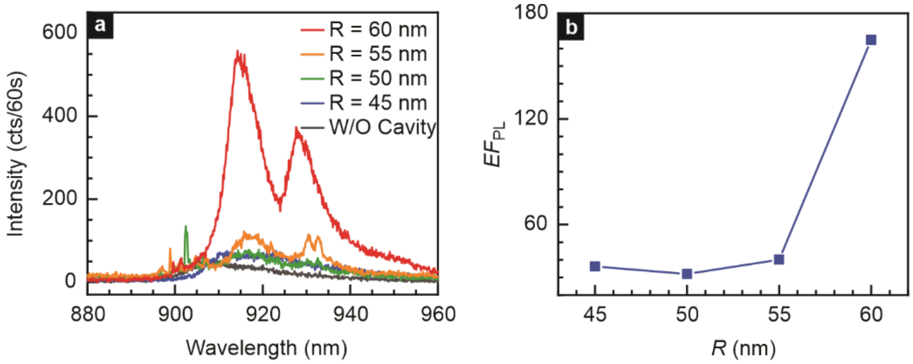

Figure S10. Photoluminescence data and enhancement factor EF_{PL} as a function of the cavity radius R for a WSe_2/$MoSe_2$ van der Waals heterostructure with a 14° twist angle. (a) PL spectra recorded for different cavity radii and in the absence of a cavity. (b) PL enhancement factor EF_{PL} as a function of R extracted from the experimental data in panel a.

S12. Discussion of the dependence of EF_{PL} on the cavity size

From the simulation results in Fig. 2b, we can see that R = 30 nm is the optimal radius to get the closest match between the interlayer exciton emission wavelength at 880 nm and the main plasmon resonance (890 nm). However, experimentally the minimal radius we can obtain reproducibly with the accessible nanofabrication tools and processing procedures is R = 35 nm. We have therefore focused on pillars with radii starting from 35 nm and an exact match between the interlayer exciton emission wavelength and the plasmon resonance wavelength cannot be achieved. It is plausible to anticipate that the smallest studied cavity with R = 35 nm gives rise to the largest enhancement factor, since the plasmon resonance is closest to the emission wavelength of the interlayer excitons. Yet, this is not the case. Instead, the largest enhancement occurs for the R = 50 nm cavity. We have identified two different reasons why for an R = 50 nm cavity the PL enhancement is larger than for the R = 35 nm cavity, even though the latter cavity has a plasmon resonance closer to the emission wavelength. We first note that the excitation wavelength of the pump laser was selected such that it coincides with the wavelength at which the absorption of the WSe_2 monolayer reaches its maximum. This wavelength equals 730 nm and is far away from any of the resonance wavelengths of the cavities with R varying between 35 and 50 nm. So, there is no advantage in this respect for a specific cavity size. However, as can be seen from the simulation results in Fig S7a of the SI, the excitation efficiency is significantly higher for the cavity with R = 50 nm. Also

the dependence of the Purcell factor on the emission wavelength favors the cavity with R = 50 nm. This is illustrated in Figure S11. Even though the main resonance peak in the Purcell factor is, as expected, far away from the wavelength of the interlayer exciton emission, there is a second maximum at shorter wavelengths. For the R = 50 nm cavity this maximum is located near 890 nm, i.e. the emission wavelength of the interlayer excitons, and this also contributes to turning the 50 nm cavity into the favored sample configuration with optimized PL enhancement factor. It is instructive to look at the distribution of the electric field intensity $|E|$ for an excitation wavelength of 890 nm (Figure S11b), where the second maximum in the wavelength dependence of the Purcell factor appears, and compare it to the distribution for 1200 nm (Figure S11c), which is close to the plasmon resonance wavelength for the R = 50 nm cavity. For excitation at 1200 nm, the optical mode is confined nearly entirely to the cavity area below the gold pillar where $|E|$ reaches values as high as 93 (Figure S11c). In contrast, for 890 nm excitation, the electric field intensity is distributed not only in that area, but also around the top of the gold pillar, albeit everywhere with a smaller magnitude. Even though we are not at the resonance wavelength, the plasmonic effect is still very significant and leads to an enormous PL enhancement.

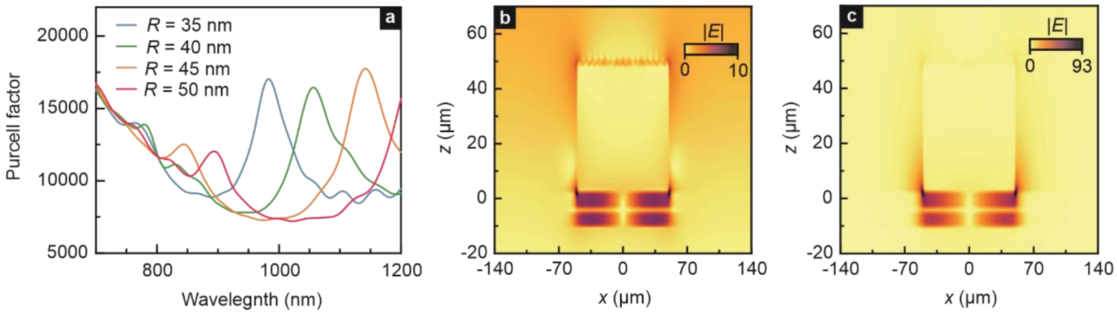

Figure S11. Simulated Purcell factor and electric field distribution. (a) Purcell factor as a function of wavelength for a cavity with R = 35, 40, 45 or 50 nm. (b) Calculated spatial distribution of the electric field intensity in the *x*-*z* plane in the vicinity of the cavity for R = 50 nm, y = 0 and an excitation wavelength of 890 nm. (c) Same as (b) but for 1200 nm excitation.

REFERENCES

(1) Wang, Q.; Wee, A. T. S. Upconversion Photovoltaic Effect of WS_2/2D Perovskite Heterostructures by Two-Photon Absorption. *ACS Nano* **2021,** *15*, 10437-10443.

(2) Wang, Q.; Guo, J.; Ding, Z.; Qi, D.; Jiang, J.; Wang, Z.; Chen, W.; Xiang, Y.; Zhang, W.; Wee, A. T. S. Fabry–Perot Cavity-Enhanced Optical Absorption in Ultrasensitive Tunable Photodiodes Based on Hybrid 2D Materials. *Nano Lett.* **2017,** *17*, 7593-7598.

(3) Wang, Q.; Zhang, Q.; Zhao, X.; Zheng, Y. J.; Wang, J.; Luo, X.; Dan, J.; Zhu, R.; Liang, Q.; Zhang, L.; Wong, P. K. J.; He, X.; Huang, Y. L.; Wang, X.; Pennycook, S. J.; Eda, G.; Wee, A. T. S. High-Energy Gain Upconversion in Monolayer Tungsten Disulfide Photodetectors. *Nano Lett.* **2019,** *19*, 5595-5603.

(4) Wang, Q.; Huang, Y. L.; Zhang, Q.; Ma, J.; Luo, X.; Li, Y.-M. Self-Energy Corrections to Zone-Edge Acoustic Phonons in Monolayer and Bilayer WS_2. *Phys. Rev. B* **2024,** *109*, L121202.

(5) Hsu, W.-T.; Zhao, Z.-A.; Li, L.-J.; Chen, C.-H.; Chiu, M.-H.; Chang, P.-S.; Chou, Y.-C.; Chang, W.-H. Second Harmonic Generation from Artificially Stacked Transition Metal Dichalcogenide Twisted Bilayers. *ACS Nano* **2014,** *8*, 2951-2958.

(6) Mennel, L.; Paur, M.; Mueller, T. Second Harmonic Generation in Strained Transition Metal Dichalcogenide Monolayers: MoS_2, $MoSe_2$, WS_2, and WSe_2. *APL Photonics* **2019,** *4*, 034404.

(7) Wang, Q.; Maisch, J.; Tang, F.; Zhao, D.; Yang, S.; Joos, R.; Portalupi, S. L.; Michler, P.; Smet, J. H. Highly Polarized Single Photons from Strain-Induced Quasi-1d Localized Excitons in WSe_2. *Nano Lett.* **2021,** *21*, 7175-7182.

(8) Shinokita, K.; Miyauchi, Y.; Watanabe, K.; Taniguchi, T.; Matsuda, K. Resonant Coupling of a Moiré Exciton to a Phonon in a $WSe_2/MoSe_2$ Heterobilayer. *Nano Lett.* **2021,** *21*, 5938-5944.

(9) Sortino, L.; Zotev, P. G.; Mignuzzi, S.; Cambiasso, J.; Schmidt, D.; Genco, A.; Aßmann, M.; Bayer, M.; Maier, S. A.; Sapienza, R.; Tartakovskii, A. I. Enhanced Light-Matter Interaction in an Atomically Thin Semiconductor Coupled with Dielectric Nano-Antennas. *Nat. Commun.* **2019,** *10*, 5119.

(10) Akselrod, G. M.; Ming, T.; Argyropoulos, C.; Hoang, T. B.; Lin, Y. X.; Ling, X.; Smith, D. R.; Kong, J.; Mikkelsen, M. H. Leveraging Nanocavity Harmonics for Control of Optical Processes in 2D Semiconductors. *Nano Lett.* **2015,** *15*, 3578-3584.

(11) Hanbicki, A. T.; Chuang, H.-J.; Rosenberger, M. R.; Hellberg, C. S.; Sivaram, S. V.; McCreary, K. M.; Mazin, I. I.; Jonker, B. T. Double Indirect Interlayer Exciton in a $MoSe_2/WSe_2$ Van Der Waals Heterostructure. *ACS Nano* **2018,** *12*, 4719-4726.

(12) Alexeev, E. M.; Ruiz-Tijerina, D. A.; Danovich, M.; Hamer, M. J.; Terry, D. J.; Nayak, P. K.; Ahn, S.; Pak, S.; Lee, J.; Sohn, J. I.; Molas, M. R.; Koperski, M.; Watanabe, K.; Taniguchi, T.; Novoselov, K. S.; Gorbachev, R. V.; Shin, H. S.; Fal'ko, V. I.; Tartakovskii, A. I. Resonantly Hybridized Excitons in Moiré Superlattices in Van Der Waals Heterostructures. *Nature* **2019,** *567*, 81-86.

(13) Seyler, K. L.; Rivera, P.; Yu, H.; Wilson, N. P.; Ray, E. L.; Mandrus, D. G.; Yan, J.; Yao, W.; Xu, X. Signatures of Moiré-Trapped Valley Excitons in $MoSe_2/WSe_2$ Heterobilayers. *Nature* **2019,** *567*, 66–70.
